# Poly-l-Lactic Acid Scaffolds Additivated with Rosmarinic Acid: A Multi-Analytical Approach to Assess The Morphology, Thermal Behavior, and Hydrophilicity

**DOI:** 10.3390/polym16121672

**Published:** 2024-06-12

**Authors:** Veronica Schiera, Francesco Carfì Pavia, Vincenzo La Carrubba, Valerio Brucato, Nadka Tz. Dintcheva

**Affiliations:** Dipartimento di Ingegneria, Università degli Studi di Palermo, Viale delle Scienze, Ed. 6, 90128 Palermo, Italy

**Keywords:** three-dimensional scaffold, poly-l-lactic acid, rosmarinic acid, thermally induced phase separation, solvent casting deposition, hydrophilicity

## Abstract

This study aims to demonstrate the possibility of incorporating a natural antioxidant biomolecule into polymeric porous scaffolds. To this end, Poly-l-Lactic Acid (PLLA) scaffolds were produced using the Thermally Induced Phase Separation (TIPS) technique and additivated with different amounts of rosmarinic acid (RA). The scaffolds, with a diameter of 4 mm and a thickness of 2 mm, were characterized with a multi-analytical approach. Specifically, Scanning Electron Microscopy analyses demonstrated the presence of an interconnected porous network, characterized by a layer of RA at the level of the pore’s surfaces. Moreover, the presence of RA biomolecules increased the hydrophilic nature of the sample, as evidenced by the decrease in the contact angle with water from 128° to 76°. The structure of PLLA and PLLA containing RA molecules has been investigated through DSC and XRD analyses, and the obtained results suggest that the crystallinity decreases when increasing the RA content. This approach is cost-effective, and it can be customized with different biomolecules, offering the possibility of producing porous polymeric structures containing antioxidant molecules. These scaffolds meet the requirements of tissue engineering and could offer a potential solution to reduce inflammation associated with scaffold implantation, thus improving tissue regeneration.

## 1. Introduction

Tissue engineering (TE) is a multidisciplinary field that integrates life sciences and engineering to develop biological substitutes that replace, repair, and enhance tissue functions [1]. Central to TE is the triad of cells, scaffolds, and growth factors. Cells play a key role in synthesizing the matrix of the new tissue, while scaffolds provide an optimal environment for cell proliferation or differentiation, and growth factors aid in the formation of new tissue [2]. A scaffold is a three-dimensional, porous structure that supports the growth, proliferation, and interconnection of cells. It also facilitates the efficient transportation of nutrients, oxygen, and waste metabolites [3,4]. The fabrication of the scaffold is crucial for the success of implants. To achieve this, the careful selection of materials and manufacturing techniques tailored to specific requirements is necessary [5,6,7,8,9]. This involves considering factors such as the shape, size, and properties of the scaffold. In particular, the materials used must be biocompatible, biodegradable with an adequate degradation time, and not release toxic degradation products [3]. Furthermore, during the design phase of a scaffold, special attention must be paid to the mechanical and physical properties of the porous matrix [4]. Once the most suitable material has been selected, the fabrication methods used to make scaffolds are varied [5,6,7,8].

Thermally Induced Phase Separation is an advanced manufacturing process known for its efficiency in producing a well-connected polymer network [9]. The process is based on a homogeneous polymer solution whose solubility equilibrium varies with temperature [10]. On cooling, phase separation occurs, leading to the formation of pores and the growth of a highly porous and interconnected structure. Precise control is achieved through carefully designed protocols that control the temperature and time during these phases [10,11]. Known for its versatility, this approach is characterized by its simplicity, speed, and adaptability, particularly in producing polymeric structures with different pore sizes and high interconnectivity [11]. Poly-l-lactic acid is a thermoplastic polymer derived from lactic acid [12]. It is produced by the ring-opening polymerization of lactide monomers and is particularly suited to the TIPS method.

PLLA is a commonly used biodegradable and biocompatible synthetic polyester in the biomedical field. It has high crystallinity, low glass transition temperature, and a high melting point. PLLA scaffolds can provide the necessary mechanical support for tissue regeneration due to their adequate mechanical properties [13]. Furthermore, the use of PLLA scaffolds has been extensively reported in the literature, in part due to its piezoelectric properties, which promote optimal tissue regeneration [14,15].

However, despite the use of biocompatible materials, scaffold implantation inevitably triggers an immune response, leading to inflammation and potential scarring that could compromise the success of the implant [16]. One of the key features of the inflammatory response is a phenomenon known as oxidative stress [17]. Oxidative stress is manifested by an overabundance of reactive oxygen species (ROS), which are characterized by an un-paired electron in their outermost orbital, making them unstable and capable of causing cellular damage by reacting readily with other molecules [18]. The use of natural antioxidants, thanks to their scavenging properties, allows the neutralization of excessive ROS, restoring the correct redox balance and reducing the inflammatory response [19,20,21].

Rosmarinic acid is a polyphenolic constituent found in many plants such as the Lamiaceae family and the subfamily Nepetoideae [22,23]. As documented, it is the ester of caffeic acid and 3,4-dihydroxyphenyllactic acid, and it has various biological effects, including antioxidant, anti-inflammatory, antibacterial, and anticancer properties, sup-ported by numerous in vivo and in vitro studies [23,24,25,26,27,28,29,30,31,32,33,34,35]. Moreover, rosmarinic acid demonstrates lipophilic characteristics, rendering it highly soluble in several organic solvents, such as ethanol (EtOH), dimethylformamide (DMF), and dimethyl sulfoxide (DMSO), while displaying poor solubility in water. The anti-inflammatory actions of RA are believed to stem from its scavenging abilities, inhibition of neutrophil activity, suppression of metalloproteinase-9 (MMP-9) activity, and modulation of the NF-jB pathway [35]. These processes suggest that RA may have potential as a treatment for inflammatory conditions through its ability to reduce inflammation and prevent tissue damage.

This information suggests that it is worth exploring the therapeutic use of RA in the development of treatments to minimize the inflammatory process caused by scaffold implantation. Of particular importance is the striking lack of studies proposing composite polymeric structures incorporating RA. To our knowledge, information about polymeric scaffolds doped with natural antioxidants is limited. Previous research by Chen et al. [36] demonstrated the possibility of incorporating other antioxidant molecules into 3D porous matrices for tissue regeneration. In their study, a 3D-printed PLLA scaffold was coated with a layer of polydopamine (PD) and then functionalized with varying concentrations of quercetin (Qu). This resulted in Qu/PD-PLLA scaffolds that showed potential for bone repair, as demonstrated by their application in MC3T3-E1 cells. Furthermore, the study conducted by Lihao et al. [37] further underscores the potential of this scaffold and antioxidant molecule approach. Utilizing 3D printing technology, they created a porous SAB-SA-Gel composite scaffold by incorporating salvianolic acid (SAB) into a matrix of sodium alginate (SA) and gelatin (Gel). This scaffold exhibited antioxidant, anti-inflammatory, and pro-angiogenic properties, reducing the expression of inflammatory factors while enhancing tissue regeneration and collagen deposition, thereby promoting diabetic wound healing. This paper aims to explore the possibility of manufacturing composite PLLA-RA scaffolds. A protocol was designed to include varying amounts of RA in PLLA scaffolds produced through the TIPS technique. The scaffolds were characterized using a range of analyses, such as gravimetric, microscopic, and spectroscopic analyses, to evaluate their morphological, thermal, and surface properties. In addition, contact angle tests were conducted to determine their hydrophilicity, providing a comprehensive assessment of their potential for tissue engineering applications.

## 2. Materials and Methods

### 2.1. Materials

Poly-l-lactic-acid (PLLA, Resomer, L 209 S, Evonik Industries, Essen, Germany; Inherent Viscosity = 2.6–3.2 dL/g) and 1,4-dioxane (Sigma-Aldrich, Munich, Germany) were used for scaffold preparation. Rosmarinic acid (RA, 96% pure, Sigma Aldrich) and Ethanol absolute anhydrous (Carlo Erba Reagents, Cornaredo, Italy) were used for ethanol/RA solution preparation.

### 2.2. Scaffold’s Preparation

PLLA scaffolds were prepared according to a previous work Lombardo et al [38]. Briefly, the polymer was dissolved in 1,4-dioxane at a concentration of 4% (*wt*/*wt*) at a temperature of 120 °C. Distilled water was then added to obtain a final dioxane/water weight/weight ratio of 87/13. Five mL of the solution, kept at 60 °C, was poured into a cylindrical high-density polyethylene sample holder (inner diameter 17.6 mm and height 35.7 mm). The sample holder was then immersed in a thermostatic water bath at 20 °C (demixing temperature) for 15 min (demixing time). At the same time, a cylindrical polytetrafluoroethylene (PTFE) coating, used to obtain a homogeneous temperature distribution in the sample holder, was pre-cooled to −20 °C. Finally, the sample holder was inserted into the PTFE cylinder, and the system was rapidly quenched by immersion in an ethyl alcohol bath at a temperature of −20 °C for at least 15 min to stop the demixing process and freeze the structure obtained. The obtained samples were washed in deionized water and dried at 60 °C to remove any remaining traces of the solvent completely. The cylindrical scaffolds were then first cut transversely into 2 mm discs and finally shaped into cylinders of 4 mm diameter and 2 mm height using a biopsy punch.

### 2.3. Rosmarinic Acid Additivation

The samples were first weighed using an ABT220-D5M (Kern, Bakersfield, CA, USA) analytical balance.

**Figure 1:**
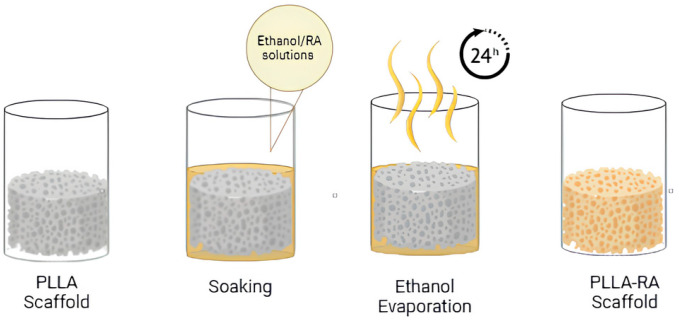
Experiment procedure scheme.

Subsequently, the weighed scaffolds were placed into a 96 multiwell plate and soaked in pure ethanol under vacuum for 2 min, to ensure complete penetration of the solvent into the pores. Once the entire surface was penetrated, the ethanol was removed. For the addition of RA, two ethanol/RA *wt*/*wt* solutions were prepared, one containing 2% *wt*/*wt* RA and the other containing 4% *wt*/*wt* RA. Then, 200 microlitres of the solution was added to each well containing scaffolds. After evaporation of the ethanol (at least 24 h), the dry samples were extracted from the well and reweighed. The procedure used is schematized in Figure 1.

### 2.4. Characterization

The percentage of additivated RA with respect to total weight was calculated as follows:

(1) $$\%RA=\frac{w_{A}-w_{B}}{w_{A}}\times 100$$

where w_A_ is the weight of the sample after RA additivation process, and w_B_ is the initial weight of the sample.

The microstructure of the scaffold was observed by Scanning Electron Microscopy (SEM) using a Philips Quanta 200 F SEM at 10 kV. The external surfaces of the samples were visualized after a gold deposition (Sputtering Scancoat Six, Edwards, Irvine, CA, USA) for 150 s.

Attenuated Total Reflectance Fourier Transform Infrared (ATR-FTIR) spectroscopy using a Spectrum One spectrometer from PerkinElmer, Waltham, MA, USA, was used to study the molecular deposition and surface structural characteristics of the material. This technique was used to investigate the vibrational modes and chemical bonds within the sample to be analyzed. For each scaffold studied, ATR-FTIR analysis was performed on both the top and bottom surfaces to determine the presence of the RA molecule. A total of 16 scans were performed at 4 cm^−1^ resolution. The ATR-FTIR spectra presented have been carefully selected based on normalized results obtained from a minimum of three samples.

The crystalline structure of RA, PLLA, and PLLA-RA scaffolds was investigated by XRD (X-ray diffraction). The measurements were carried out through a Panalytical X‘Pert Powder Diffractometer with 2θ angle ranging from 5° to 35°,with a step angle and a step time of 0.1° and 10 s, respectively. The voltage was 40 kV, and the tube current was 30 mA.

The samples were analyzed calorimetrically using a DSC Setaram 131 evo. Pure PLLA, RA powder, additivated PLLA-RA samples, and an RA film obtained via solvent casting were analyzed. Each sample was subjected to two heating scans. The samples were carefully weighed and placed in aluminum crucibles for analysis, and the following thermal protocol was applied: first heating from 25 °C to 220 °C at 10 °C/min, held at 220 °C for 10 min, cooling to 50 °C at 10 °C/min, held at 50 °C for 10 min, and second heating from 25 °C to 220 °C at 10 °C/min. Melting enthalpies and temperatures were determined using data processing Calisto software–2.0.

The static contact angle test was performed using an FTA 1000 (First Ten Ångstroms, Cambridge, UK) instrument with distilled water (DW) as the liquid. Specifically, a drop of DW (~4 μL) was dropped onto the scaffold, and images were taken 10 s after DW deposition.

## 3. Results

### 3.1. Gravimetrical Analysis

**Figure 2:**
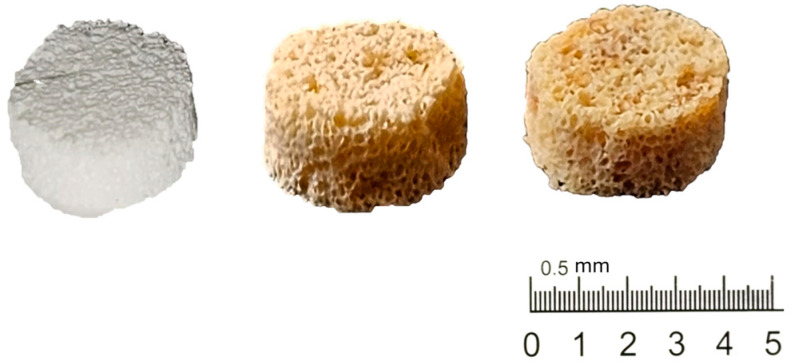
Digital images of samples, right to left: PLLA, PLLA-RA 2%, and PLLA-RA4%.

In this study, we used the solution deposition method starting with two solutions at 2% and 4% *wt*/*wt*, as reported in Section 2. Figure 2 shows the picture of pure PLLA and RA additivated samples. A clear change in the color of the scaffold can be attributed to the presence of RA in the samples.

In order to evaluate the presence of RA incorporated into the scaffold, 5 samples of each type were weighed dry before and after the deposition of RA. The obtained data are presented in Table 1. A significant amount of biological molecules of RA are clearly incorporated into the polymeric structure, leading to a substantial increase in weight.

### 3.2. Morphology Evaluation

**Figure 3:**
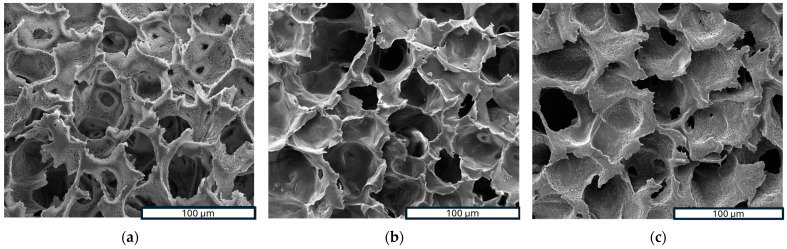

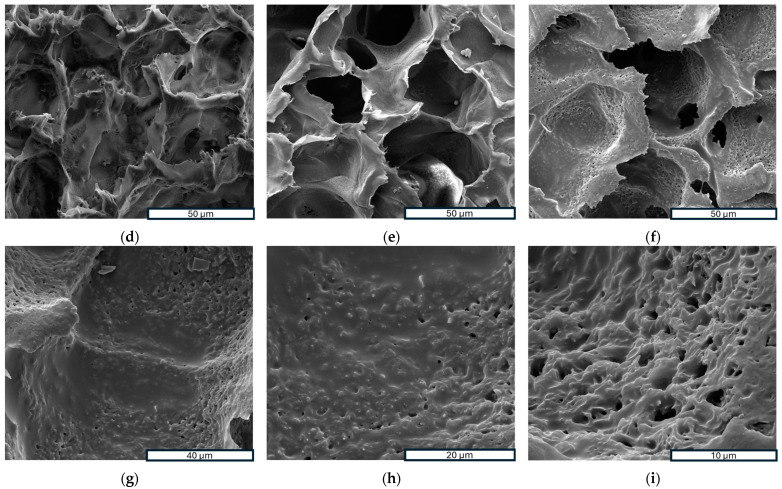
SEM images: (**a**–**c**) porous structure analysis of PLLA, PLLA-RA 2%, PLLA-RA at 600×; (**d**–**f**) porous structure analysis of PLLA, PLLA-RA 2%, PLLA-RA at 1200×; (**g**–**i**) porous structure analysis of PLLA-RA 4% at 2600×, 5000×, and 10,000×.

Scanning Electron Microscopy (SEM) is a powerful analytical technique widely utilized for examining the surface morphology and microstructure of scaffolds at a microscopic level. In this study, SEM was employed to investigate the morphology of samples. Figure 3a–f show SEM images of three investigated scaffolds at different magnifications, while Figure 3g–i provide a closer look at the high magnification of a PLLA-RA 4% scaffold. As noticeable, the dimensions of pores could be estimated ranging from 50 to 70 μm with a good interconnectivity.

The images in Figure 3a–f show that the pores’ morphology remained unchanged despite the presence of RA. Moreover, the micrographs at high magnification of PLLA-RA 4% samples revealed the presence of an RA layer (see Figure 3g–i) at the level of the pore’s surfaces. Additionally, the presence of the RA layer can be observed in Figure 3e, although it was less pronounced.

### 3.3. Spectroscopy Evaluation

**Figure 4:**
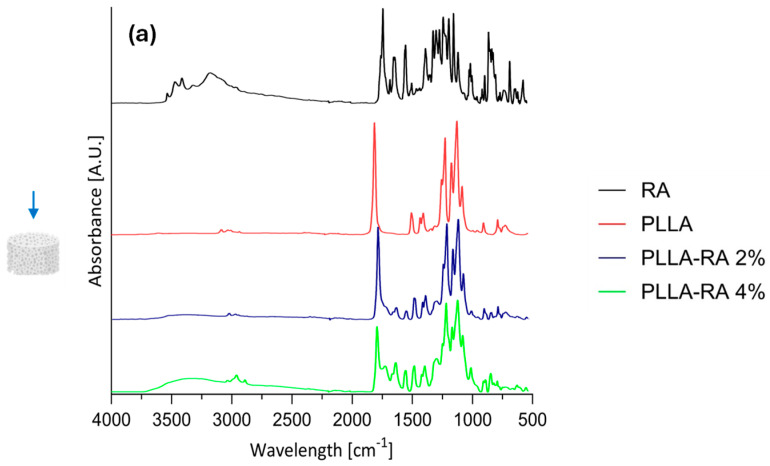

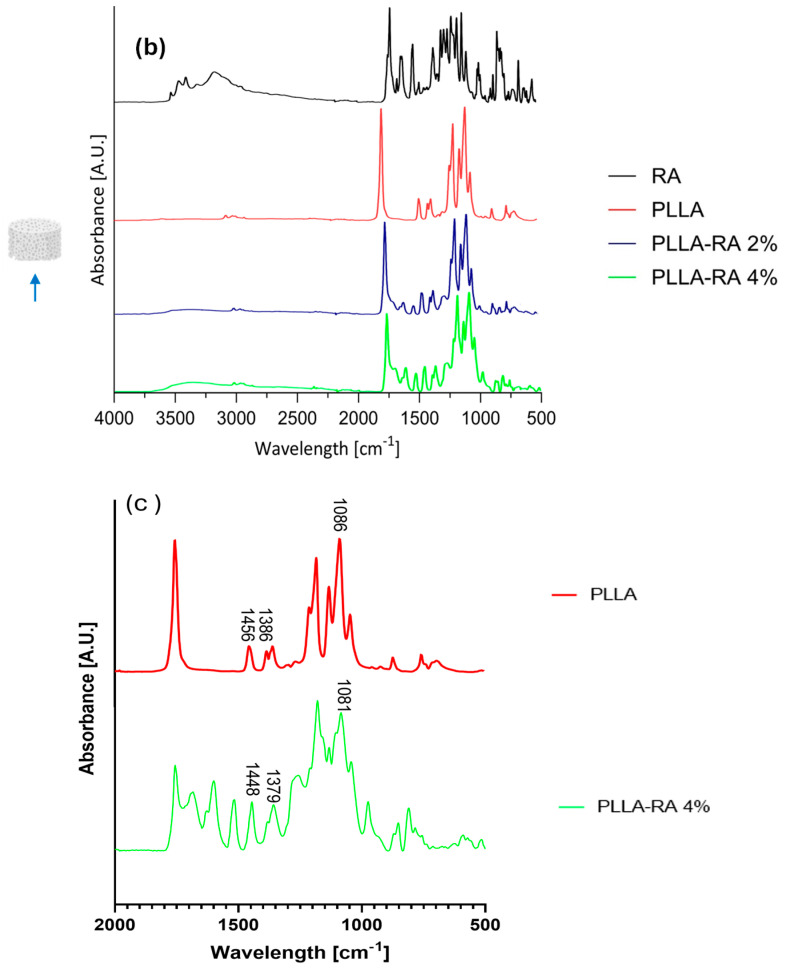
ATR-FIIR spectra: (**a**) RA powder and on top surfaces of PLLA, PLLA-RA 2%, PLLA-RA 4%; (**b**) RA powder and on bottom surfaces of PLLA, PLLA-RA 2%, PLLA-RA 4%; (**c**) PLLA and PLLA-RA 4%.

Results of Attenuated Total Reflectance Fourier Transform Infrared Analysis (ATR-FTIR) analysis on RA powder and PLLA, PLLA-RA 2%, and PLLA-RA 4% scaffolds are shown in Figure 4a,b for top and bottom surfaces.

In addition, according to the literature, Table 2 contains the assignments of characteristic peaks of both PLLA [39] and RA [40]. As expected, the pure PLLA sample, on both top and bottom surfaces, showed no peaks in the spectral region between 3500 and 3000 cm^−1^, while RA itself showed several peaks in this region related to phenolic −OH stretching, occurring at c.a. 3500 cm^−1^, and C−H stretching, occurring at the frequencies above 3000 cm^−1^. Further, within the range at 1700–1000 cm^−1^, distinct peaks appeared in RA spectra, specifically, one at 1700 cm^−1^ corresponding to the stretching vibration of >C=O, followed by peaks around 1605 and 1520 cm^−1^ indicating stretching of the aromatic ring. In addition, two other signals appeared at 1360 cm^−1^ and 1180 cm^−1^ due to O−H and C−O stretching, respectively.

In the spectra of PLLA-RA 2% and PLLA-RA 4% samples, changes in peaks in the regions around 3500–3000 cm^−1^ and 1700–1000 cm^−1^ were observed. In particular, PLLA-RA 2% and PLLA-RA 4% showed a small shoulder around 3500–3000 cm^−1^. Furthermore, PLLA showed various intrinsic peaks in the range of 1700–1000 cm^−1^, and as noticeable, the spectra of PLLA-RA 2% and 4% showed more complex peaks in this region.

Interestingly, some specific characteristic peaks in PLLA shifted to lower frequencies due to the presence of RA molecules. Specifically, the −C−O− stretch at 1086 cm^−1^ in the spectrum of PLLA shifted to 1081 cm^−1^ in the spectrum of PLLA/RA 4%, and the −CH and CH_3_ stretches at 1386 cm^−1^ and 1456 cm^−1^ shifted to 1379 cm^−1^ and 1448 cm^−1^, respectively. Further, small shifts for the bands at ca. 3000 and 2945 cm^−1^ attributed to the stretching of −CH groups were also noticed. All these changes suggest that in the samples containing RA, the interactions between the RA biomolecules and PLLA scaffold structure occurred.

### 3.4. Diffractometric Analysis

**Figure 5:**
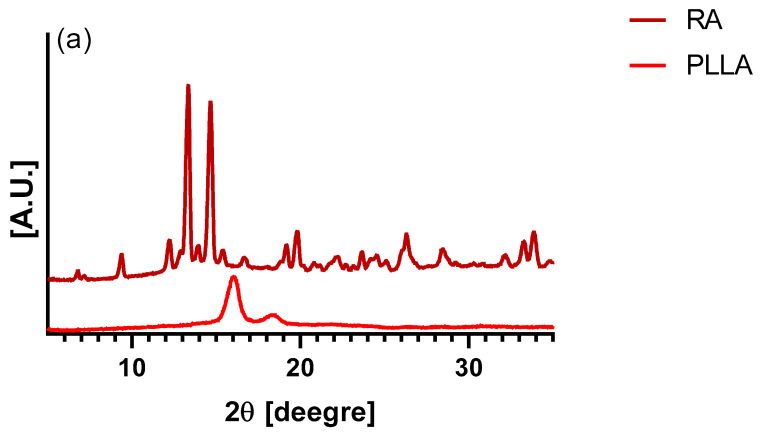

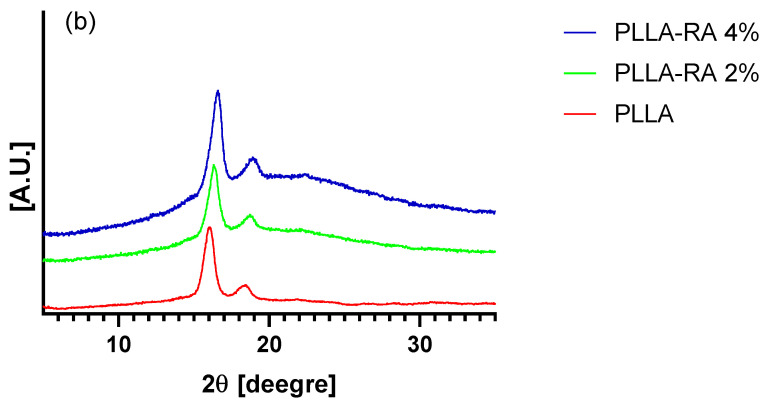
XRD trace of (**a**) PLLA and RA samples, and (**b**) PLLA, PLLA-RA 2%, PLLA-RA 4%.

XRD (X-ray diffraction) analysis of the PLLA samples and rosmarinic acid are shown in Figure 5a. Pure PLLA pattern presents two typical peaks located at 15.6 and 18.3 degrees, which are associated with the crystalline component of the biopolymer [41]. Figure 5b shows the XRD patterns of PLLA and PLLA-RA samples. It is easy to notice that the RA peaks are totally absent in the composite samples, whereas the PLLA peaks are well noticeable. Finally, a significant increase in the amorphous halo can be noticed when increasing the RA content.

### 3.5. Thermal Analysis

**Figure 6:**
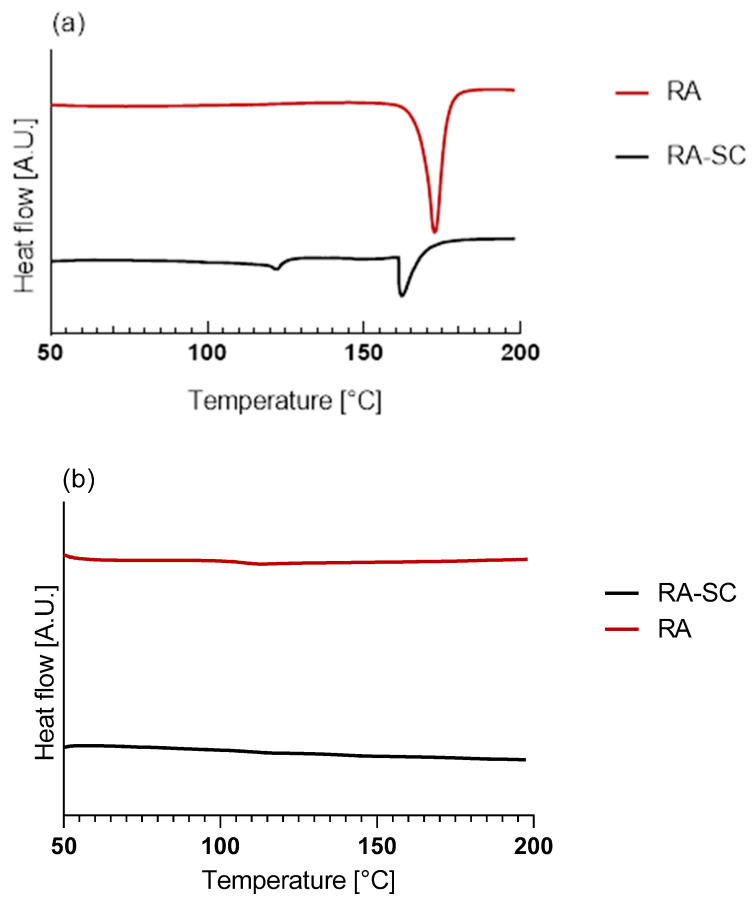

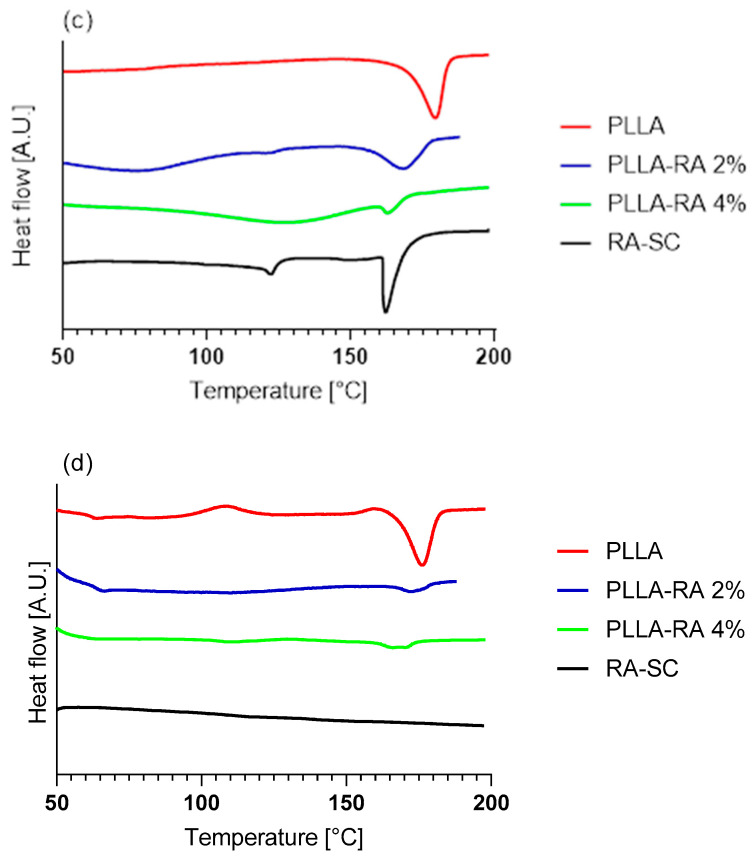
Thermograms of Differential Scanning Calorimetry (DSC) analysis: (**a**) first heating of RA powder and RA-SC; (**b**) second heating of RA powder and RA-SC; (**c**) first heating of PLLA, PLLA-RA 2%, PLLA-RA 4%, and RA-SC; (**d**) second heating of PLLA, PLLA-RA 2%, PLLA-RA 4%, and RA-SC.

Since the RA is additivated to the scaffolds through a solvent casting procedure, a thin film of RA, obtained with the same technique, was prepared, analyzed, and compared to RA powder. Figure 6a,b illustrate, respectively, the thermograms of the first and second heating of the RA powder and RA-solution-casted film (RA-SC).

The data obtained from the tests are shown in Table 3. It can be observed that there was a large difference in terms of melting enthalpy and temperature between the two samples. No peaks were detected in the second heating, due to the degradation of RA over 200 °C, which agrees with the value reported in the literature [42].

The thermograms of PLLA, PLLA-RA 2%, PLLA-RA 4%, and RA-SC of the first and second heating are shown in Figure 6c,d. From the thermograms, it can be noticed that the melting peaks of the composite scaffolds appear very different with respect to the peak of pure PLLA. Specifically, PLLA-RA 2% presents a broader peak, whereas the PLLA-RA 4% peak appears very small. The data analyses reveal a concentration-dependent decrease in melting enthalpies and temperatures of PLLA when increasing RA content. As a matter of fact, in the PLLA-RA-2%, the melting enthalpy decreases from 72 to 39 J/g, whereas the melting temperature drops from 181 to 174 °C. A further decrease is observed in PLLA-RA-4% samples for both investigated parameters. The obtained data are displayed in Table 3.

### 3.6. Surface Analysis of Hydrophilicity

**Figure 7:**
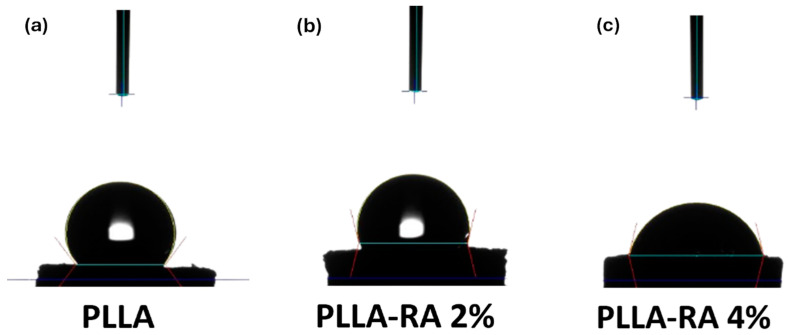
Water contact angle (WCA) image of (**a**) PLLA; (**b**) PLLA-RA 2%; (**c**) PLLA-RA 4%.

Figure 7 shows the water contact angle (WCA) values obtained with distilled water on PLLA, PLLA-RA 2%, and PLLA-RA 4% samples. The test was performed on three samples of each type to assess the change in the hydrophilicity of the scaffold.

As can be noticed from Figure 7, the water contact angle decreases when increasing RA concentration, highlighting that the presence of RA induces the hydrophilicity change. Interestingly, the water contact angles of PLLA samples remain unchanged over time. For example, immediately after water deposition, the value is 128.40° ± 1.5°. After one minute, it decreases slightly to 128.28° ± 1.3°, and after 5 min, it further decreases to 127.86 ± 1.2°. This suggests that the PLLA scaffold maintains this hydrophobicity in limited time (ca. 5 min). The water angle contact of PLLA-RA 2% and PLLA-RA 4% samples is 103.96° ± 1.2° and 76.51° ± 1.0°, respectively. After 1 min, the water drop disappears. The water absorption is in favor of RA presence, and the measurement cannot be performed.

## 4. Discussion

In this work, the solution deposition method was employed to incorporate an antioxidant molecule into polymeric scaffolds produced via TIPS. Scaffolds, with a diameter of 4 mm and a thickness of 2 mm, were obtained and characterized. Ascribing to the presence of RA, the color of the whole surface of the scaffolds changed from white to yellow. Moreover, when the concentration of RA increased, the pigmentation of the samples was more evident. A gravimetric analysis revealed that the percentual of RA incorporated in the samples doubled when passing from 2% to 4% solution. The data obtained from the analysis show that samples of PLLA-RA 2% and PLLA-RA 4% contained high concentrations of the antioxidant molecule, approximately 47.7%, and 81.9%, respectively.

An analysis of SEM micrographs revealed the presence of an interconnected porous network in the samples. As known, the pore dimension is widely regarded as one of the most important requirements of a scaffold for tissue regeneration. A study by Bergonzi et al. showed that increasing the concentration of antioxidants, particularly vitamin E, led to changes in pore size and a wider range of pore sizes in the scaffold [43]. The deposition method used in this study to incorporate natural antioxidant molecules into the scaffolds preserved the interconnectivity and maintained the original pore size. Moreover, as the concentration of antioxidants on the scaffold increased, a visible layer of RA became more evident at the level of the surface of the structure. All things considered, the Thermally Induced Phase Separation method with solvent casting deposition allowed the production of 3D porous structures capable of accommodating high concentrations of antioxidant molecules while precisely controlling pore size and interconnectivity.

ATR-FTIR analysis was conducted to determine the presence of RA molecules on both the top and bottom surfaces of the scaffold. All obtained results suggest that the RA solution penetrated and permeated the entire three-dimensional polymeric structure. The neat RA showed typical intrinsic peaks in the regions around 3500–3000 cm^−1^ and 1700–500 cm^−1^ and assignment to the presence of phenolic and carboxylic functionalities [40]. The neat PLLA sample shows typical peaks in 1700–1000 cm^−1^ according to the literature [39].

The spectra of both PLLA-RA samples showed an evident presence of phenolic functionalities and more complex peaks around 1700–1000 cm^−1^ in comparison to PLLA sample. These changes were attributed to the presence of RA molecules, especially in the PLLA-RA 4% sample, due to the lower amount of PLLA, which is only 18% of the total weight.

Furthermore, it can be assumed that the biomolecule interacts physically with the polymer through the formation of hydrogen bonds; this kind of interaction is confirmed by several papers in which polymer/polyphenol systems were analyzed through the same technique [44,45,46].

These considerations confirm the presence of RA molecules and the occurrence of the interactions between PLLA and RA molecules, according to the SEM images.

In order to establish if the presence of RA could have effects on the scaffold crystallinity, XRD spectra were carried out. Surprisingly, the XRD patterns of PLLA-RA scaffold did not show the RA peaks. This observation leads one to state that the crystallization of RA was completely inhibited, and it was present on the scaffold in a totally amorphous state. The presence in these samples of a more evident amorphous halo with the respective neat PLLA supports this hypothesis.

Calorimetric analyses substantially confirm the integration of RA and its distribution throughout the PLLA-RA scaffolds. The RA powder shows a melting enthalpy about three times that of the RA-SC film, and additionally, RA shows higher fusion temperatures than the RA-SC one. These results show that after solubilization of the powder in ethanol, the formation of crystalline structures by the RA molecule is disfavored, and as expected, the RA molecules are organized in a predominantly amorphous state with low crystalline content, in comparison to RA powder. Once the RA powder sample reaches the upper temperature of 200 °C, the molecules probably undergo irreversible degradation. The second rise of the RA powder and RA-SC samples shows small humps at a temperature of about 120 °C probably due to the reorganization of the decomposition products of the RA molecule.

However, it seems that RA exhibits a different behavior, in terms of crystallization kinetics during the additivation process, as it is present in the scaffolds only in amorphous state (as evidenced by XRD analyses).

PLLA scaffolds show a reduction in the fusion enthalpy of second heating of fusion of about 20.8% in comparison to the first fusion enthalpy, while the temperature of fusion changes from 181 °C to 177 °C.

The samples containing 2% and 4% PLLA-RA do not show two distinct peaks for RA and PLLA, but only one peak. As the amount of RA increases, a decrease in their melting temperatures compared to pure PLLA is observed. In the case of 4% PLLA-RA, this value is very similar to the RA value. Additionally, a decrease in melting enthalpies compared to pure PLLA is also observed. Specifically, considering the melting enthalpy and temperature values of 4% PLLA-RA, that are 6 J/g and 165 °C, respectively, it is possible to hypothesize that the RA addition protocol, adopted here, influences the PLLA crystallinity. Indeed, even assuming that the melting peak is exclusively due to PLLA and recalculating the enthalpy value normalized to the PLLA weight (i.e., 18% of sample weight is PLLA), a melting enthalpy of 33 J/g can be calculated, that is significantly lower than the experimentally measured value of 68 J/g for pure PLLA.

Therefore, it is not easy to establish the specific contributions of PLLA and RA to the melting peaks and/or enthalpies, since the RA is in the amorphous state in the composite samples, and moreover, it interacts with PLLA.

Hydrophilicity is considered to play an important role in the interaction between the scaffold and the tissue. For tissue engineering applications, good scaffold hydrophilicity is required for cell adhesion and proliferation. Several studies reported in the literature have shown that the use of polyphenolic coatings was able to improve the hydrophilicity of the scaffold surfaces [47].

The WCA values of the PLLA scaffold were revealed at three different times (immediately after water drop deposition and after 1 and 5 min of deposition). The result suggests that the PLLA sample maintained in the time its hydrophobic nature. Different results were obtained for the samples after the surface modification by RA. In PLLA-RA 2% and PLLA-RA 4% scaffolds, the WCA values decreased when increasing the RA concentration. Moreover, it was noticed that the droplet deposited on the samples in a few seconds appeared distributed over the entire surface. These phenomena could be explained by the presence of polyphenolic compounds in the RA according to the data found in the literature [47].

All obtained results suggest that the considered ad hoc protocol allows the successful production of PLLA scaffolds, incorporating large amounts of RA molecules. As previously stated, our main objective was to produce a composite scaffold and effectively incorporate RA molecules, without specifically examining the biological activities of RA. As a result, this particular topic will be the center of attention for our upcoming study, which will serve as a natural extension of the current work.

## 5. Conclusions

In our study, we successfully incorporated rosmarinic acid, a natural antioxidant, into Poly-l-Lactic acid scaffolds. To achieve this, we introduced a novel protocol to incorporate a natural biomolecule, which is soluble in organic solvents, into the polymeric scaffolds produced via TIPS. This approach is not only cost-effective but also customizable with different biomolecules. The presence of RA molecules in the whole scaffold structures was confirmed by ATR-FTIR analysis. The resulting scaffolds showed well-defined pore networks with good interconnectivity, even in those containing different amounts of rosmarinic acid (up to 81.9% of RA). Notably, these scaffolds not only exhibit a favorable morphology but also excellent hydrophilicity, meeting the requirements for tissue engineering. The water contact angle of the samples decreased from 128.40° ± 1.5° to 76.51° ± 1.0°. The DSC and XRD analyses suggest that the RA was in amorphous state, and due to the interactions between the PLLA and RA, the overall crystallinity of the scaffolds decreased.

Our focus on composite PLLA-RA scaffolds yielded promising results, indicating that incorporating natural antioxidant molecules into polymeric structures could be a potential solution to mitigate implant-associated inflammation, opening new avenues for future development in this field.

## Figures and Tables

**Table 1:** Weight percentages (%) of PLLA and RA in the different samples.

Sample Code	% PLLA Component	% RA Component
PLLA	100	0
PLLA-RA 2%	56.23±1.31	44.77±1.31
PLLA-RA 4%	18.03±3.62	81.97±3.62

**Table 2:** FTIR spectra of PLLA and RA peak band assignments.

Sample	Peak Position (cm^−1^)	Assignment	Ref.
PLLA	3507	−OH stretch	[39]
	2993, 2943	−CH stretch	
	1746	−C=O carbonyl stretch	
	1450	−CH_3_ bend	
	1381, 1358	−CH− deformation including symmetric and asymmetric bend	
	1265	−C=O bend	
	1183, 1128, 1086	−C−O− stretch	
	1044	−OH bend	
	925, 868	−C−C stretch	
RA	>3000	−CH stretch −OH stretching of the phenolic groups	[40]
		COOH-carboxylic groups stretching	
	1750–1725	Ester groups	
	1725, 1700, 1395 ± 55	Carboxylic groups	
	1605, 1520, 1445	Aromatic ring stretching	
	1360	−OH stretch	
	1180	C−O stretch	

**Table 3:** Melting enthalpies and temperatures of first and second heating.

Sample	${∆H}_{fus}I\;Heat$ (J/g)	Temp.fus.I Heat (°C)	${∆H}_{fus}II\;Heat$ (J/g)	Temp.fus.II Heat (°C)
RA powder	135.4±12.3	172	−	−
RA−SC	35.09	162	−	−
PLLA	69.43±2.6	181	55.70±1.7	177
PLLA−RA 2%	29.36±5.0	174	12.65±2.0	172
PLLA−RA 4%	6.67±0.2	163	6.46±0.7	166

− Not determined.

## Data Availability

Data are contained within the article.
